# Polymorphisms in Fibronectin Binding Proteins A and B among *Staphylococcus aureus* Bloodstream Isolates Are Not Associated with Arthroplasty Infection

**DOI:** 10.1371/journal.pone.0141436

**Published:** 2015-11-25

**Authors:** Emily M. Eichenberger, Joshua T. Thaden, Batu Sharma-Kuinkel, Lawrence P. Park, Thomas H. Rude, Felicia Ruffin, Nina J. Hos, Harald Seifert, Siegbert Rieg, Winfried V. Kern, Steven K. Lower, Vance G. Fowler, Achim J. Kaasch

**Affiliations:** 1 Division of Infectious Diseases & International Health, Department of Medicine, Duke University School of Medicine, Durham, NC 27710, United States of America; 2 Duke Global Health Institute, Duke University, Durham, NC 27710, United States of America; 3 Institute for Medical Microbiology, Immunology and Hygiene, University of Cologne, Goldenfelsstraße 19-21, 50935 Cologne, Germany; 4 German Centre for Infection Research (DZIF), Bonn-Cologne, University of Cologne, Albertus-Magnus-Platz, 50923 Cologne, Germany; 5 Division of Infectious Diseases, Department of Medicine II, University Medical Center Freiburg, Hugstetter Straße 55, 79106 Freiburg, Germany; 6 Division of Natural and Mathematical Sciences, Ohio State University, Columbus, OH 43210, United States of America; Universitätsklinikum Hamburg-Eppendorf, GERMANY

## Abstract

**Background:**

Nonsynonymous single nucleotide polymorphisms (SNPs) in fibronectin binding protein A (*fnbA*) of *Staphylococcus aureus* are associated with cardiac device infections. However, the role of *fnbA* SNPs in *S*. *aureus* arthroplasty infection is unknown.

**Methods:**

Bloodstream *S*. *aureus* isolates from a derivation cohort of patients at a single U.S. medical center with *S*. *aureus* bacteremia (SAB) and prosthetic hip or knee arthroplasties that were infected (PJI, n = 27) or uninfected (PJU, n = 43) underwent sequencing of *fnbA* and *fnbB*. A validation cohort of *S*. *aureus* bloodstream PJI (*n* = 12) and PJU (*n* = 58) isolates from Germany also underwent *fnbA* and *fnbB* sequencing.

**Results:**

Overall, none of the individual *fnbA* or *fnbB* SNPs were significantly associated with the PJI or PJU clinical groups within the derivation cohort. Similarly, none of the individual *fnbA* or *fnbB* SNPs were associated with PJI or PJU when the analysis was restricted to patients with either early SAB (i.e., bacteremia occurring <1 year after placement or manipulation of prostheses) or late SAB (i.e., bacteremia >1 year after placement or manipulation of prostheses).

**Conclusions:**

In contrast to cardiac device infections, there is no association between nonsynonymous SNPs in *fnbA* or *fnbB* of bloodstream *S*. *aureus* isolates and arthroplasty infection. These results suggest that initial steps leading to *S*. *aureus* infection of cardiovascular and orthopedic prostheses may arise by distinct processes.

## Introduction

*Staphylococcus aureus* is a leading cause of prosthetic joint infection (PJI) [1,2]. A hallmark of *S*. *aureus* PJI is the biofilm, a sessile community of bacteria fixed in an extracellular matrix of DNA, proteins and polysaccharides. Biofilms promote device-associated infection and are highly resistant to host defenses and antimicrobials [3]. Integral to the initiation of *S*. *aureus* biofilm formation are fibronectin binding protein A and B (FnBPA, FnBPB), members of the family of Microbial Surface Components Recognizing Adhesive Matrix Molecules (MSCRAMMs) [4–7].

Our lab previously demonstrated that specific, non-synonymous single nucleotide polymorphisms (SNPs) in *fnbA* are associated with cardiac device infection (CDI) and stronger binding to fibronectin as determined by atomic force microscopy *in vitro* [8,9]. This association between *fnbA* SNPs and CDI was recently validated [10]. However, it is unknown whether this association extends to *S*. *aureus* infections of other prosthetic devices, such as arthroplasties. The current study investigates potential associations between variation in *fnbA* and *fnbB* and the likelihood of arthroplasty infection in a patient with *S*. *aureus* bacteremia (SAB).

## Materials and Methods

### Source of *S*. *aureus* Isolates

This study involved human participants and was approved by the IRB Committees at Duke University Medical Center, University of Cologne, and University Medical Center at Freiburg. Written consent to participate in the study was received from all participants. Adult patients with SAB were eligible for inclusion in the current analysis if they had a hip or knee arthroplasty present at time that *S*. *aureus* was isolated from ≥1 blood culture. Patients with polymicrobial infections were excluded (n = 1). Isolates from eligible patients were divided into two groups: a *derivation cohort* and a *validation cohort*.

*Derivation cohort*: Derivation cohort isolates were obtained from the *S*. *aureus* Bacteremia Group (SABG). SABG is an ongoing prospective cohort study at Duke University Medical Center (Durham, NC). The SABG database consists of prospectively ascertained clinical data, patient DNA, and bloodstream bacterial isolates from more than 2,000 consecutive consenting patients with SAB since 1994. SABG patients are evaluated prospectively during their hospital stays for source of infection, presence of prosthetic devices, hemodialysis dependency, clinical signs of *S*. *aureus* infection, length of stay, discharge status, and clinical outcome [11].

*Validation Cohort*: The validation cohort was derived from the INSTINCT (Invasive *S*. *aureus* Infection Cohort) study [12]. INSTINCT is conducted at two German centers: University of Cologne and University Medical Center Freiburg. INSTINCT has collected clinical data and bacterial isolates from more than 1,000 consecutive patients since 2006.

### Clinical Definitions

Two clinical categories were identified: prosthetic joint infected (PJI) and prosthetic joint uninfected (PJU). PJI was defined as isolation of *S*. *aureus* from the arthroplasty site during the initial episode of SAB. PJU was defined as no evidence of device infection at the time of the initial episode of SAB, retention of the arthroplasty, and no evidence of infected arthroplasty or recurrent infection 12 weeks after the initial onset of SAB. Bacterial isolates were obtained from the initial blood culture specimens to allow for better comparison of PJI and PJU (e.g., there were no isolates from uninfected joints by definition). Early infection was defined as SAB occurring <1 year after prostheses implantation or manipulation. Late infection was defined as SAB occurring >1 year after placement or manipulation of prostheses.

### Amino Acid Polymorphisms in FnBPA and FnBPB

Each *S*. *aureus* isolate underwent genomic DNA extraction using an Ultraclean Microbial DNA Isolation kit (MO BIO). The DNA then underwent high-fidelity PCR amplification of *fnbA* and *fnbB*. The resulting fragments were sequenced in the forward and reverse directions to obtain the full sequence length of the *fnbA* and *fnbB* binding regions [9]. Their nucleotide and predicted amino acid sequences (FnBPA and FnBPB, respectively) were compared with the respective reference sequences obtained from *S*. *aureus* NCTC 8325–4 by the ClustalW method using DNASTAR and Vector NTI [9]. These experiments were repeated for the isolates from the external validation cohort [10] by investigators (EME, BSK, THR) who were blinded to the clinical designation (i.e., PJI vs. PJU) of the isolates.

### Genotyping of Bacterial Isolates by *spa* Typing

Genomic DNA was prepared as described above. Each DNA isolate underwent high-fidelity PCR with specifically designed forward and reverse primers to amplify the *spa* region [13]. The resulting PCR fragment underwent DNA purification and sequencing at the Duke University sequencing laboratory. The Ridom SpaServer (www.spaserver.ridom.de) was used to map the resulting sequences to the corresponding *spa* type and clonal complex.

### Fibronectin Binding Assay

An assay with slight modification from that of Peacock *et al*. [14] was used to determine *S*. *aureus* binding to fibronectin *in vitro*. Briefly, 96-well BD Falcon plates were coated with 100 μL human fibronectin (10 μg/mL, Sigma) overnight at 4°C. The plates were washed with phosphate-buffered saline (PBS) and then blocked with 100 μL of 2% bovine serum albumin solution for 1 hour at 37°C. Wells were washed and 100 μL bacteria collected from overnight TSB cultures and adjusted in PBS to optical density 1.0 at 600 nm (corresponding to 10^7^ cells/mL) were added in quadruplicate and incubated for 2 hours at 37°C. The wells were washed again with 100 μL PBS. Bacteria were fixed with 100 μL of 25% formaldehyde solution for 10 minutes. Then 100 μL of a 0.5% crystal violet (Sigma) solution was added to each well for 1 minute. Finally, 100 μL dimethyl sulfoxide was added to each well before absorbance was read at 620 nm using a Multiskan Ascent 354 Plate Reader (Thermo Labsystems). Absorbance values were then expressed as percentage of the values obtained for *S*. *aureus* 8325–4 on the same plate. Each strain underwent a total of three fibronectin binding assays, the results of which were averaged.

### Biofilm Assay

The biofilm assay was performed as described by Sharma-Kuinkel *et al*. [15] and Beenken *et al*. [16] with slight modifications. Briefly, 96-well BD Falcon plates were coated with 200 μL of 20% human plasma (Sigma) in carbonate buffer (pH 9.6, 0.05M) overnight at 4°C. The plasma was then discarded from each well. Next, 200 μL bacteria grown overnight in TSB-NaCl-glucose broth and then adjusted to optical density 0.05 at 600 nm was added in quadruplicate to the wells and incubated for 24 hours at 37°C. The cultures were discarded from the wells and the plate washed with 200 μL PBS. Biofilms were fixed using 200 μL of 100% ethanol for 2 minutes at room temperature. The wells were then stained with 100 μL of 0.41% crystal violet in 12% ethanol for 2 minutes at room temperature, then washed with 200 μL PBS. Biofilms were dried at room temperature overnight and then solubilized with 100 μL of 100% ethanol for 10 minutes at room temperature. Absorbance was read at 620 nm using a Multiskan Ascent 354 Plate Reader (Thermo Labsystems). Each plate included a positive control *S*. *aureus* strain known to form biofilm (UAMS-1), another *S*. *aureus* strain that weakly forms a biofilm (8325–4), and a negative control (medium only) [16,17]. The capacity to form a biofilm was compared against these strains. Bacterial isolates were deemed to have a proteinaceous matrix when they satisfied two conditions: 1) no biofilm formed when grown in the presence of 2 μg/mL proteinase K [18], and 2) dissolution of biofilm when 2 μg/mL proteinase K was added to 36-hour biofilms and incubated for 24 hours. The biofilm matrix composition of one PJI isolate and one PJU isolate were tested.

### Quantitative PCR

To evaluate the possibility that variable expression of *fnbA* could be associated with risk of PJI, a random sample of isolates (7 PJI, 7 PJU) from the derivation cohort underwent quantitative PCR to determine expression levels of *fnbA*. Isolates with multiple SNPs and isolates with few to no SNPs were equally represented within each group. The 18 isolates along with positive control (8325–4) were grown overnight in 10 mL liquid TSB media at 37°C. Bacteria collected from the overnight cultures and adjusted to an OD 0.1 at 600 nm were added to 10 mL fresh TSB liquid media and incubated at 37°C for 4 hours. RNA was then extracted from each isolate using the RNeasy Mini Kit (Qiagen). RNA concentrations were measured using a NanoDrop (Thermo Scientific) to measure the ratio of UV absorption at 260 nm and 280 nm. Next, cDNA was synthesized via RT-PCR from each RNA template using the BIO-RAD iScript cDNA Synthesis kit (170–8890). Then 2 μL cDNA was added to 96-well PCR plates in duplicate. Specially designed forward and reverse *fnbA* primers and the BIO-RAD SYBR Green Supermix (152–7560) were added to the cDNA as detailed by the BIO-RAD qPCR protocol. The plate was then placed in the BIO-RAD qPCR/Real Time PCR machine, and the fluorescence intensity was measured following each amplification cycle.

### Western Ligand Affinity Blotting

Western ligand affinity blotting was performed as previously described [4], with modifications. Briefly, overnight cultures were diluted 1:50 in TSB medium and grown at 37°C with shaking for 4 hours. Cells were washed and surface-associated protein extracts were prepared through resuspension in PBS media containing 20 μg/mL lysostaphin (Sigma), 20 μg/mL DNAse (New England Biolabs), 1 mM phenylmethanesulfonylfluoride (PMSF, Thermo Scientific), and 1:100 dilution of a protease inhibitor cocktail (Sigma, P2714). Cell extracts were incubated at 37°C for 30 minutes and spun at 12,000 x g for 1 minute at 4°C. The protein concentration in the supernatant was determined with a BCA Protein Assay Kit (Pierce), and 20 μg of each sample was separated via sodium dodecyl sulfate-polyacrylamide gel electrophoresis (SDS-PAGE). FnBPs were detected by incubation for 1 hour with biotinylated human fibronectin (50 μg/mL) in PBS containing 0.1% Tween 20 (PBST). An EZ-Link sulfo-*N*-hydroxysuccinimide-LC biotinylation kit (Pierce) was used to biotinylate human fibronectin (Sigma). After washing with PBST, membranes were incubated for 1 hour with streptavidin-peroxidase conjugate (Roche; 1:3000 dilution). Finally, membranes were developed with the ECL Western blotting system (Pierce). A *S*. *aureus fnbA fnbB* double mutant [19] was used as a negative control.

### Statistical Analysis

Data were presented as frequency counts and corresponding percentages for categorical factors, and means for continuous variables [13]. Fisher’s Exact Test and unpaired t-tests with an alpha = 0.05 were used to calculate significance using Microsoft Excel and JMP Pro 10.2. Given that multiple comparisons (i.e., multiple SNPS) were tested, a false discovery rate procedure was applied to the raw p-values to correct for false associations [20]. Raw p-values are displayed in Tables 1, 2 and 3, with exception as noted in Tables 2 and 3. A p-value <0.05 was considered statistically significant [13].

**Table 1:** Demographic and clinical characteristics of patients in the derivation cohort with *S*. *aureus* bacteremia and infected or uninfected prostheses.

CHARACTERISTICS	PROSTHETIC JOINT INFECTION (n = 27)	PROSTHETIC JOINT UNINFECTED (n = 43)	P-value
**SEX**			
Female	18 (67%)	25 (58%)	0.62
**RACE**			
Black	5 (19%)	13 (30%)	0.40
White	22 (81%)	30 (70%)	
**COMORBIDITIES**			
Diabetes mellitus	6 (22%)	19 (44%)	0.08
Hemodialysis	1 (4%)	6 (14%)	0.24
Cancer	4 (15%)	5 (12%)	0.73
**TIMING OF INFECTION**			
Early	10 (37%)	3 (7%)	0.002
Late*	17 (63%)	40 (93%)	
**METHICILLIN RESISTANCE**			
	12 (44%)	27 (69%)	0.15
**MEAN AGE**			
	61 (range 20–81)	66 (range 33–88)	0.15
**DEVICE**			
Hip	17 (52%)	24 (56%)	0.62
Knee	10 (37%)	18 (42%)	0.80
Both	0	1 (2%)	1.00

*Late infection is defined as bloodstream infection occurring >1 year after the prostheses was implanted or surgically manipulated.

**Table 2:** Single Nucleotide Polymorphisms (SNPs) in fibronectin binding protein A (*fnbA)* in the derivation cohort, external validation cohort, and late *S*. *aureus* bacteremia (SAB) group. In the derivation cohort, no SNPs occurred with greater frequency in the prosthetic joint infection group (PJI) relative to the uninfected prosthetic joint group (PJU). In the external validation cohort, one SNP (S839N) was significantly associated with the PJU group, though when the two cohorts were combined the S839N association did not reach statistical significance (p = 0.22). Late SAB was defined as bacteremia occurring >1 year after placement or manipulation of prostheses, and here contains data from both the derivation and external validation cohorts. In the late SAB group, no SNPs occurred with greater frequency in PJI or PJU.

NONSYNONYMOUS SNP	DERIVATION COHORT			EXTERNAL VALIDATION COHORT			LATE *S*. *AUREUS* BACTEREMIA		
	PJI (n = 27)	PJU (n = 43)	P-value	PJI (n = 12)	PJU (n = 58)	P-value	PJI (n = 27)	PJU (n = 80)	P-value
**REPEAT 1**									
T539K	1	0	0.39	0	2	1.00	1	0	0.25
**REPEAT 2**									
G563E	3	6	1.00	1	11	0.68	4	17	0.58
**REPEAT 5**									
E652D	2	5	0.70	0	10	0.19	4	15	0.78
V668I	2	0	0.15	-	-		1	0	0.25
**REPEAT 6**									
S678I	1	0	0.15	0	2	1.00	0	0	1.00
V698I	4	8	0.76	0	14	0.11	4	20	0.42
K703E	27	40	0.28	2	24	0.19	16	47	1.00
**REPEAT 7**									
D723G	4	7	1.00	0	15	0.06	4	19	0.42
**REPEAT 8**									
G741S	-	-	-	1	0	0.17	1	0	0.25
N750D	1	0	0.39	0	2	1.00	0	0	1.00
**REPEAT 9**									
P779T	-	-	-	0	7	0.34	0	1	1.00
H782Q	1	0	0.39	0	2	1.00	0	2	1.00
K786N	3	6	0.39	0	13	0.11	1	13	0.11
N788D	-	-	-	0	2	1.00	0	2	1.00
K797E	-	-	-	0	3	1.00	0	2	1.00
**REPEAT 10**									
H804Q	2	2	0.64	0	2	1.00	1	2	1.00
H818Q	1	0	0.39	0	2	1.00	0	0	1.00
T826N	2	0	0.15	0	3	1.00	1	0	0.25
**REPEAT 11**									
S839N	6	10	1.00	0	20	0.01*	5	25	0.23
N846S	-	-	-	0	2	1.00	0	1	1.00
S847I	-	-	-	0	1	1.00	0	1	1.00
K857H/Q	0	1	1.00	0	2	1.00	0	2	1.00
Q861H	0	1	1.00	0	2	1.00	0	2	1.00

*When false discovery rate control is applied, this raw p-value no longer maintains statistical significance (p = 0.22).

**Table 3:** Single Nucleotide Polymorphisms (SNPs) in fibronectin binding protein B (*fnbB)* in *fnbB-*containing isolates. No SNP was associated with the prosthetic joint infected (PJI) or uninfected (PJU) isolates in the derivation cohort, external validation cohort, or late *S*. *aureus* bacteremia (SAB) group. Late SAB was defined as SAB occurring >1 year after placement or manipulation of prostheses.

NONSYNONYMOUS SNP	DERIVATION COHORT			EXTERNAL VALIDATION COHORT			LATE *S*. *AUREUS* BACTEREMIA		
	PJI (n = 18)	PJU (n = 29)	P-value	PJI (n = 11)	PJU (n = 55)	P-value	PJI (n = 18)	PJU (n = 61)	P-value
**REPEAT 1**									
E458D	3	4	1.00	5	28	0.76	5	27	0.28
E462D	0	1	1.00	1	2	0.43	1	2	0.54
H463K	3	3	0.66	5	27	1.00	6	26	0.59
T465E	3	3	0.66	5	27	1.00	7	27	0.79
E468D	1	0	0.38	1	15	0.27	2	15	0.33
E470D	3	3	0.66	4	25	0.75	5	19	1.00
F471I	3	3	0.66	5	28	0.76	7	27	0.79
P475Q	1	2	1.00	0	5	0.58	1	3	1.00
T483N	0	1	1.00	1	1	0.32	1	2	0.54
**REPEAT 2 or 3**									
A506V	2	3	1.00	1	8	1.00	3	11	1.00
A510V	1	1	1.00	-	-	-	0	0	1.00
T513I	5	7	1.00	7	28	0.75	7	27	0.79
I514V	3	1	0.15	2	13	1.00	4	13	1.00
**REPEAT 4**									
H530L	1	0	0.38	0	2	1.00	1	0	0.23
**REPEAT 5**									
T554I	1	1	1.00	-	-	-	0	1	1.00
D565E	1	3	1.00	4	24	0.75	5	25	0.41
V570L	1	1	1.00	-	-	-	0	1	1.00
I581V	5	8	1.00	8	31	0.53	9	26	0.60
**REPEAT 6**									
P601L	-	-	-	0	2	1.00	0	2	1.00
E602K	-	-	-	2	10	1.00	2	9	1.00
**REPEAT 7**									
N618Y	2	4	1.00	4	19	1.00	5	16	1.00
H619Q	2	4	1.00	4	21	1.00	5	19	1.00
G633S	1	0	0.38	1	0	0.17	2	0	0.05*
S645T	-	-	-	1	0	0.17	1	0	0.23
**REPEAT 9**									
H696Q	3	5	1.00	0	10	0.19	2	14	0.34
N700K	3	4	1.00	0	5	0.58	2	9	1.00
N702D	9	19	0.37	5	32	0.53	9	40	0.27
K711E	2	4	1.00	1	7	1.00	3	9	1.00
**REPEAT 10**									
Q718H	1	2	1.00	0	5	0.58	0	0	1.00
I723L	-	-	-	0	2	1.00	0	1	1.00
H732Q	2	2	0.63	2	9	1.00	3	11	1.00
T740N	3	2	0.36	8	27	0.34	8	26	1.00
**REPEAT 11**									
N753S	1	0	0.38	-	-	-	0	0	1.00
N760S	5	8	1.00	1	7	1.00	4	8	0.45
S761G	1	0	0.38	-	-	-	0	0	1.00
V761I	1	0	0.38	-	-	-	0	0	1.00
E765V	1	0	0.38	-	-	-	0	0	1.00
Q771K	1	1	1.00	3	9	0.42	3	7	0.69
G774V	2	0	0.14	0	2	1.00	2	1	0.13
H775Q	1	1	1.00	2	7	0.65	3	5	0.37
S761I	-	-	-	0	2	1.00	0	2	1.00
T781N	-	-	-	0	1	1.00	0	0	1.00

*When false discovery rate control is applied, this raw p-value no longer maintains statistical significance (p = 1.00).

## Results

### Clinical Characteristics of Derivation Cohort

The derivation cohort contained *S*. *aureus* bloodstream isolates from 70 unique patients (27 PJI; 43 PJU). Patients in the two clinical groups were similar with respect to sex, race, age, medical comorbidities, infection with MRSA, and joint location (Table 1). Significantly more patients in the PJI group had early infection (10/27 vs 3/43; p = 0.002), likely because of an increased risk of arthroplasty infection in the perioperative period.

### *spa* Typing

A total of 24 different *spa* types were identified in the derivation cohort. Of these, *spa* types 2 (24 isolates; 34.3%), 12 (8 isolates; 11.4%), and 18 (6 isolates; 8.6%) were most common. The *spa* types were mapped to the broader categories of clonal complexes (CC). The most prevalent CC in the derivation cohort was CC5 (24 isolates, 34.3%) followed by CC30 (14 isolates, 20.0%).

A total of 38 different *spa* types were identified in the validation cohort. Of these, *spa* type 2 (8 isolates; 11.4%), *spa* type 91 (7 isolates; 10%), and *spa* type 3 (5 isolates; 7.1%) were the most common. When these *spa* types were mapped to corresponding CC, CC5 (15 isolates, 21.4%), CC7 (7 isolates, 10%), CC15 (6 isolates, 8.6%), and CC45 (6 isolates, 8.6%) predominated.

**Fig 1:**
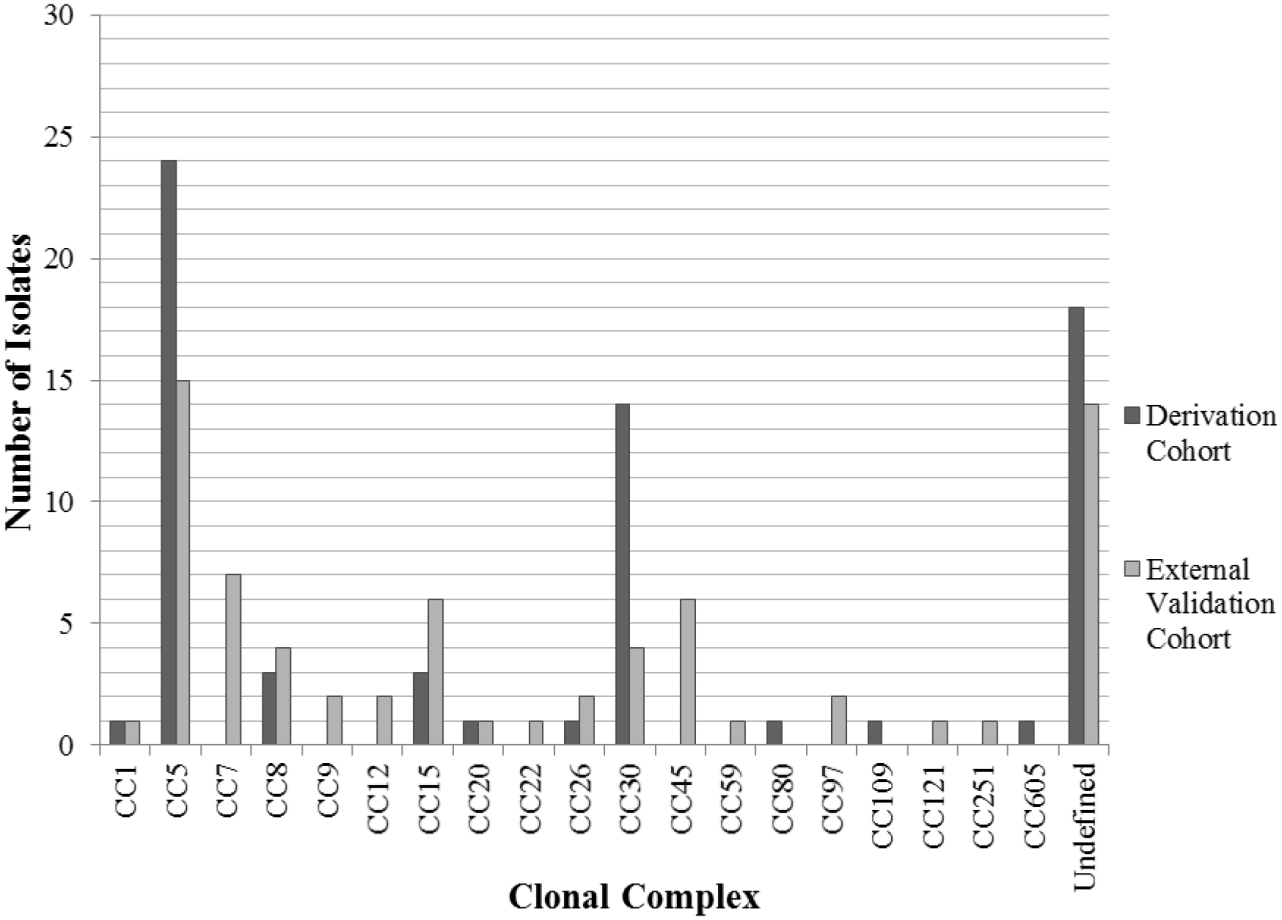
Distribution of clonal complexes amongst *S*. *aureus* bloodstream isolates in the derivation and external validation cohorts. Clonal Complex 30 (CC30) was significantly more common in the derivation cohort (p = 0.01), while CC7 and CC45 were significantly more common in the external validation cohort (p = 0.01 and p = 0.03, respectively).

CC30 was significantly more common in the derivation cohort as compared to the validation cohort (14/70 [20%] isolates vs. 4/70 [5.7%] isolates, p = 0.01). Conversely, CC7 and CC45 were significantly more common in the validation cohort (CC7: 7/70 [10%] isolates vs. 0 isolates, p = 0.01; CC45: 6/70 [8.6%] isolates vs 0 isolates, p = 0.03) (Fig 1). Overall, there was no significant association between any of the CC and clinical group (i.e., PJI or PJU).

### Sequencing of *fnbA*

**Fig 2:**
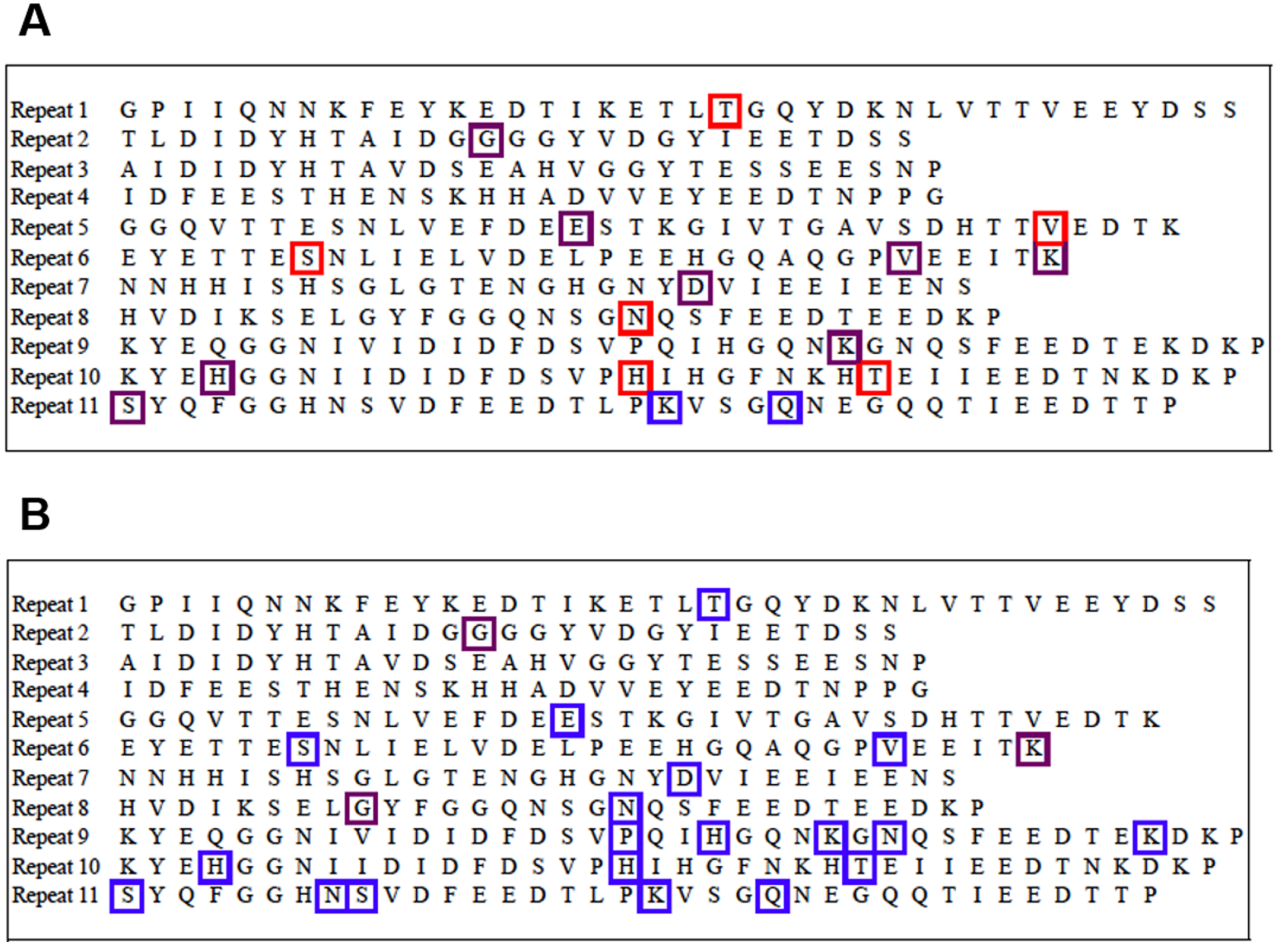
Single Nucleotide Polymorphisms (SNPs) in the binding regions (Repeats 1–11) of fibronectin binding protein A (FnBPA) in the derivation cohort (A) and external validation cohort (B). Red boxes indicate SNPs that were only present in isolates from the prosthetic joint infection group (PJI), blue boxes indicate SNPs that were only present in the uninfected (PJU) group, and purple boxes indicate SNPs that were present in both PJI and PJU isolates.

All 70 isolates in the derivation cohort contained the *fnbA* gene, as determined by PCR. A total 17 different non-synonymous SNPs were found in the fibronectin binding regions of *fnbA*. Fifteen of the 17 SNPs were found in the PJI isolates and 10/17 SNPs were found in the PJU isolates (88.2% vs 58.8%; p = 0.11) (Fig 2A). In the derivation cohort, no SNP was significantly more common in either of the clinical groups (Table 2). This includes the three SNPs that were previously associated with cardiac device infection [9,10]–E652D (p = 0.70), H782Q (p = 0.39), and K786N (p = 0.39). The lack of association between SNP frequency and clinical group remained when bacteria were analyzed within their individual clonal complexes (data not shown).

### Sequencing of *fnbB*

**Fig 3:**
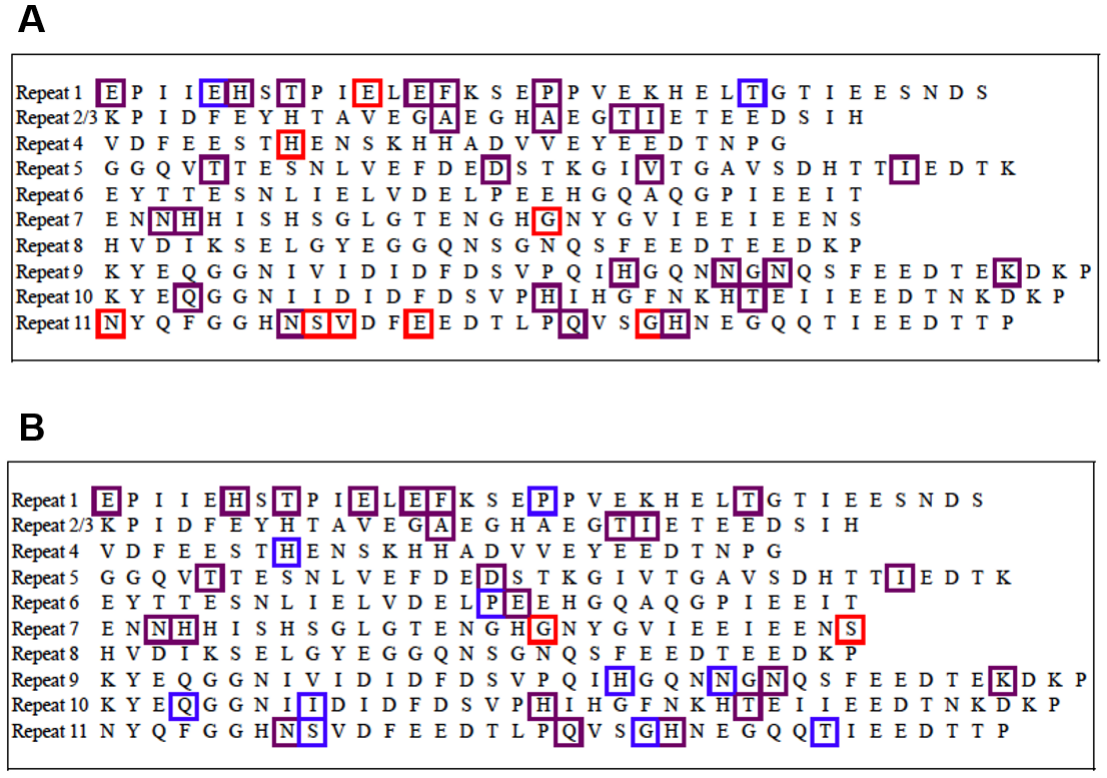
Single Nucleotide Polymorphisms (SNPs) in the binding regions (Repeats 1–11) of fibronectin binding protein B (FnBPB) in the derivation cohort (A) and the external validation cohort (B). Red boxes indicate SNPs that were only present in isolates from the prosthetic joint infection group (PJI), blue boxes indicate SNPs that were only present in the uninfected (PJU) group, and purple boxes indicate SNPs that were present in both PJI and PJU isolates.

A total 18 (66.7%) PJI isolates and 29 (67.4%) PJU isolates in the derivation cohort possessed *fnbB* (p = 1.00), as determined by PCR. Overall, 36 different non-synonymous SNPs were found in the binding regions of the 47 isolates containing this gene (Fig 3A). Thirty-four of these 36 SNPs (94.4%) were found in the PJI group, and 28/36 SNPs (60.0%) were found in the PJU group (p = 0.08). None of the individual SNPs were significantly more common in either of the clinical groups (Table 3). This lack of association between SNP frequency and clinical group remained when bacteria were analyzed within their individual clonal complexes (data not shown).

### Fibronectin Binding and Biofilm Formation

**Fig 4:**
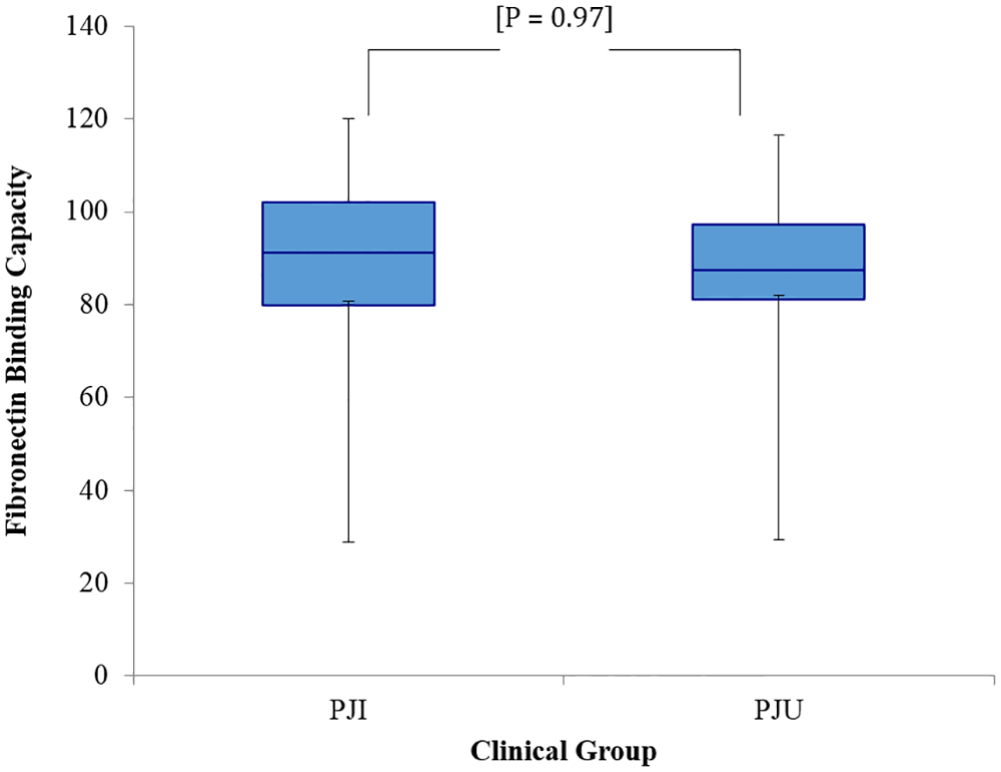
Comparison of the fibronectin binding capacity of *S*. *aureus* isolates from prosthetic joint infection (PJI) and uninfected prosthetic joint (PJU) groups. Values were calculated as percentage capacity to bind fibronectin relative to *S*. *aureus* control strain 8325–4. Box ends represent the 25^th^ and 75^th^ percentiles, and whisker ends represent the minimum and maximum. There was no difference in fibronectin binding capacity between isolates in the PJI and PJU groups.

**Fig 5:**
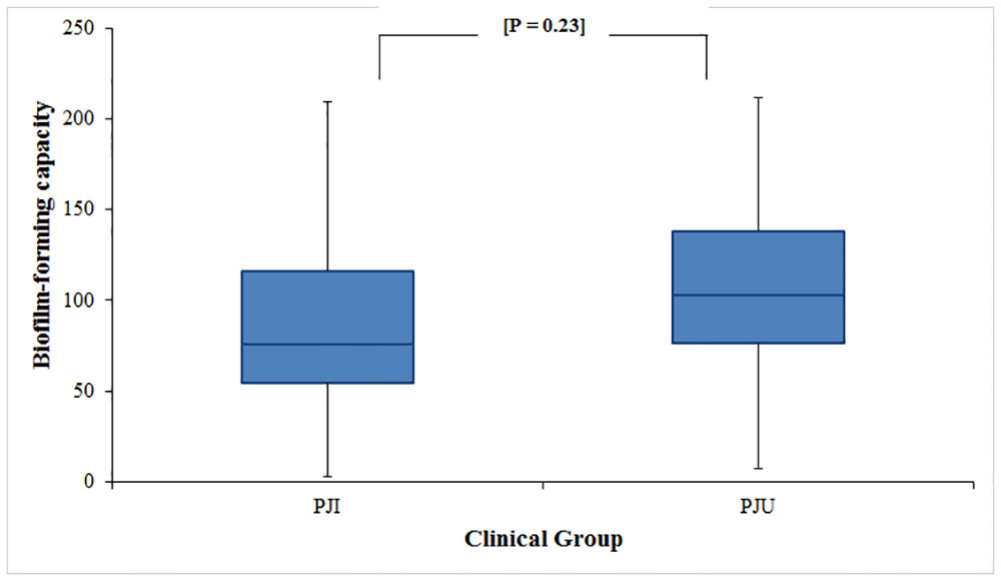
Comparison of the biofilm-forming capacity of *S*. *aureus* isolates from prosthetic joint infection (PJI) and uninfected prosthetic joint (PJU) groups. Values were calculated as percentage capacity to form biofilms relative to *S*. *aureus* control strain UAMS-1. Box ends represent the 25^th^ and 75^th^ percentiles, and whisker ends represent the minimum and maximum. There was no difference in biofilm-forming capacity between isolates in the PJI and PJU groups.

Bacterial isolates within the two clinical groups of the derivation cohort exhibited a similar capacity for fibronectin binding (mean fibronectin binding: PJI 87.1% vs PJU 87.3%; p = 0.97) (Fig 4) and ability to form biofilm (mean biofilm formation: PJI 90.0% vs PJU 104.7%; p = 0.23) (Fig 5). Representative PJI and PJU isolates were tested and found to have proteinaceous biofilm matrices. Neither clinical grouping, particular *fnbA* SNPs, nor particular *fnbB* SNPs were significantly associated with reduced fibronectin binding or capacity to form biofilm. In addition, increased fibronectin binding was not significantly correlated with degree of biofilm formation (Pearson correlation coefficient r = 0.06; S1 Fig).

### *fnbA*/FnBPA Expression

**Fig 6:**
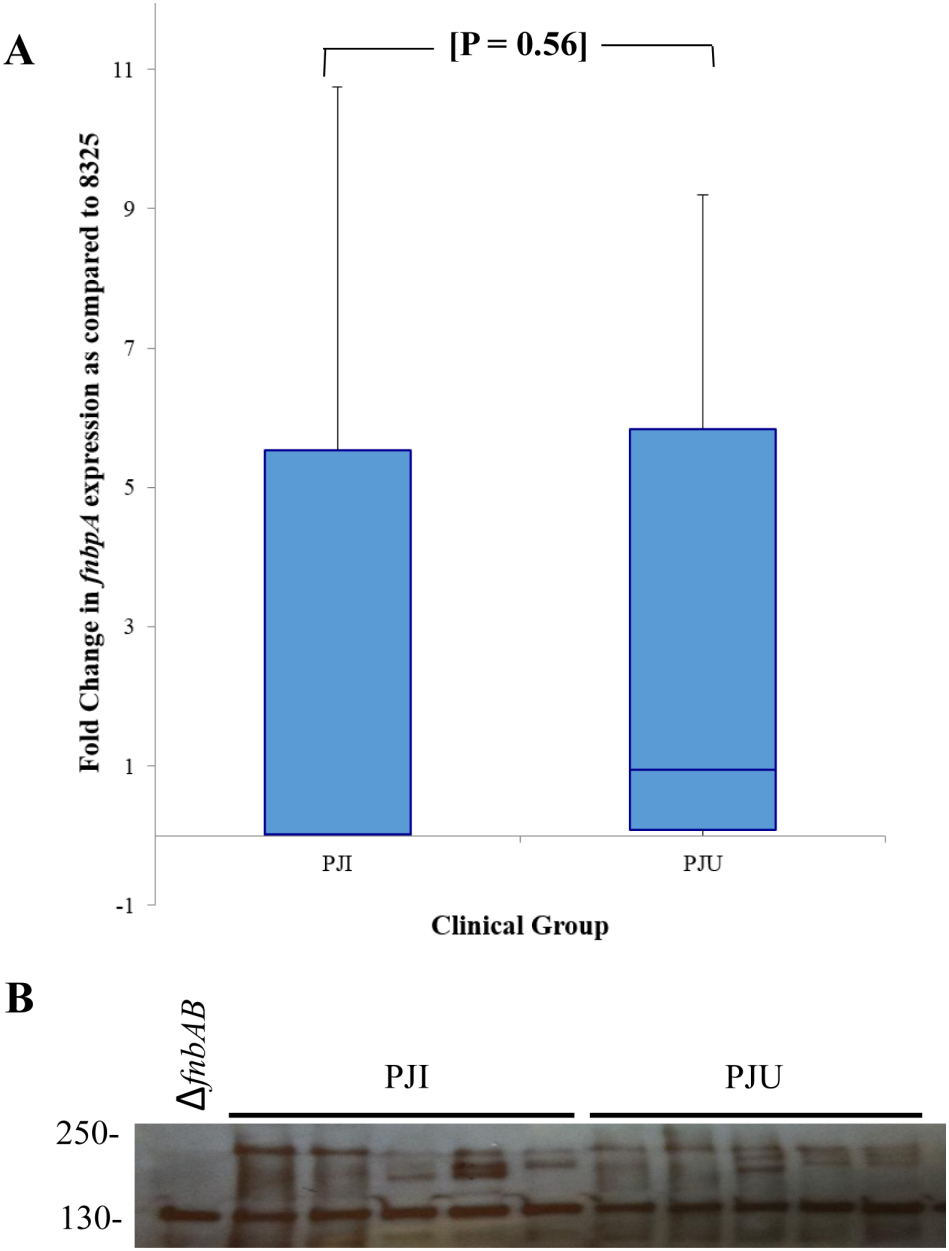
Fibronectin binding protein A (*fnbA)* RNA expression (A) and fibronectin binding proteins (FnBPs) expression (B) in randomly selected *S*. *aureus* bloodstream isolates from patients with infected (PJI) and uninfected (PJU) prosthetic hip or knee joints. (A) RNA expression is represented as percent *fnbA* expression relative *to S*. *aureus* control 8325–4. Box ends represent the 25^th^ and 75^th^ percentiles, and whisker ends represent the minimum and maximum. There is no significant difference in *fnbA* expression between the two groups. (B) Fibronectin-binding protein expression was determined with Western ligand affinity blotting. There is no major difference in expression between the two clinical groups. Protein size (kilodaltons) is displayed and the double mutant *fnbAB* is included as a control.

Twenty percent (14 isolates) of the derivation cohort was randomly selected to assess level of *fnbA* RNA expression. The absorbance reading values obtained through RT-PCR were reported as fold-change in RNA expression relative to control 8325–4. The level of *fnbA* expression was not significantly different between the PJI and PJU isolates (p = 0.56) (Fig 6A). In addition, a Western ligand affinity blot using biotinylated human fibronectin was used to assess the expression of *S*. *aureus* fibronectin-binding proteins in a subset of isolates (5 PJI, 5 PJU). The bacterial isolates were chosen at random, and the PJI and PJU isolates were similar with respect to antibiotic susceptibility (i.e., MSSA vs. MRSA), timing of bacteremia (i.e., early vs. late) and site of prostheses. Though there was variation in fibronectin-binding protein expression, no clear difference between the PJI and PJU isolates was observed (Fig 6B). This assay measures all fibronectin-binding proteins and is not specific to FnBPA and FnBPB.

### Validation Cohort

The validation cohort contained 70 patients: 12 PJI (17.1%), and 58 PJU (82.9%). Twenty-two distinct SNPs in *fnbA* identified in the validation cohort isolates were present in at least one of the PJU isolates, while only 3 were present in PJI isolates (22/22 [100%] in PJU vs 3/22 [13.6%] in PJI; p = 0.001) (Fig 2B). Of the 22 SNPs identified, one SNP (S839N) was more common in the PJU group as compared to the PJI group (p = 0.01) (Table 2). After adjustment for multiple comparisons, however, the statistical significance of this finding was lost (p = 0.22). When the derivation and external validation cohorts were combined, the S839N SNP was not associated with the PJU group (p = 0.09).

Of the 70 validation cohort isolates, 66 contained *fnbB* (94.3%). The presence of *fnbB* was not associated with orthopedic device infection (66.7% PJI vs 67.4% PJU, p = 1.00). Among isolates with *fnbB* in the validation cohort, 35 distinct SNPs were found in the fibronectin-binding region of *fnbB*. Twenty-five SNPs (71.4%) were found in the PJI group and 33 (9.3%) in the PJU group (p = 0.02) (Fig 3B). There were no *fnbB* SNPs that occurred with greater frequency in the PJI group versus the PJU group (Table 3). When the derivation and external validation cohorts were combined, there were similarly no *fnbB* SNPs associated with PJI or PJU.

### Early Versus Late PJI

Given that early PJI and late PJI are likely to involve different mechanisms of pathogenesis (e.g., early PJI from a primary surgical site infection; late PJI from hematogenous seeding), we evaluated *fnbA* and *fnbB* SNPs within these two clinical groups separately. Interestingly, an *fnbB* SNP (G633S) was associated with PJI in the late SAB group when the derivation and validation cohorts were combined (PJI: 2/18 [11%]; PJU: 0/61 [0%]; p = 0.05). However, the statistical significance of this relationship was not maintained after adjustment for multiple comparisons (p = 1.00) (Table 3). There were no other significant associations between *fnbB* SNPs and PJI or PJU in the early or late SAB groups. No *fnbA* SNPs were associated with either PJI or PJU in either the early or late SAB groups (Table 2).

## Discussion

The biological basis of *S*. *aureus* prosthetic device infections is incompletely understood. The current investigation evaluates whether the association that exists between *fnbA* SNPs and cardiac device infection extends to arthroplasties. Our study demonstrates that, unlike cardiac devices, *S*. *aureus* infection of arthroplasties is not associated with polymorphisms in the fibronectin binding protein genes, increased fibronectin binding, or biofilm formation capacity. This finding has several important implications.

In contrast to what our lab [9] and others [10] reported in *S*. *aureus* cardiac device infections, no SNPs in *S*. *aureus fnbA* were associated with infections of arthroplasties. Of the 17 distinct SNPs found in the *fnbA* binding region of the derivation cohort, no single mutation occurred with greater frequency in the PJI group versus the PJU group. In particular, the three SNPs that were previously shown to be associated with cardiac device infections—E652D, H782Q, and K786N –were all present in both the derivation and validation cohorts though not statistically associated with PJI. There are a few possible explanations for this intriguing difference between *S*. *aureus* PJI and cardiac device infection. First, cardiac devices such as pacemakers and cardioverter defibrillators are endovascular. Thus, they are subject to high levels of blood flow, exposure to various blood components and host proteins, and hemodynamic shear stress. In contrast, arthroplasties are extravascular, exist within a “sanctuary space”, and are exposed to a different array of proteins and flow stress dynamics.

Second, cardiac devices are coated shortly after implantation with a proteinaceous fibrin sheath composed of host proteins including fibronectin and fibrinogen [3,21–24]. The deposition of these host proteins on the endovascular components of cardiac devices significantly enhances adherence of *S*. *aureus* to the device [21]. By contrast, arthroplasties do not develop a fibrin sheath [25]. They are instead coated with osteopontin, bone sialoprotein and α_2_ HS-glycoprotein [25]. It is believed that these proteins accumulate because they contain polyacidic sequences (Asp-Gly-Asp) that promote cell adhesion [25]. Recent studies characterizing bone sialoprotein binding protein (*bbp*) of *S*. *aureus* have speculated that this MSCRAMM may be an important molecular player in the pathogenesis *S*. *aureus* osteomyelitis and PJI [26,27]. Through riboprinting, Campoccia et al. found that *bbp*, the gene encoding for bone sialoprotein binding protein in *S*. *aureus*, was highly prevalent (94%) in *S*. *aureus* isolates causing orthopedic device infections. Additionally, studies in rabbit models have suggested that *bbp* is generally only harbored by virulent *S*. *aureus* strains [28].

Third, cardioverter defibrillators, pacemakers, and their leads contain different materials than those in arthroplasties. The pacemaker generator and lithium battery are encased in a metal, typically titanium, while the leads are coated with polyurethane or silicone rubber [29,30]. By contrast, the femoral component of hip arthroplasties usually contains steel, cobalt-chromium alloys, or titanium alloys, while the cup component may contain ceramic, cobalt-chromium or polytetrafluoroethylene (PTFE, Teflon) [31].

Given the distinct properties of cardiac devices and arthroplasties, we speculate that the association between cardiac device infections and the presence of *fnbA* SNPs may be due at least in part to the unique conditions associated with such endovascular infections that are absent in orthopedic implants. Additionally, we propose that there may be other bacterial and/or host factors that predispose certain patients to PJI. Further research is underway to elucidate which factors those may be.

The current study also considered the potential role of *fnbB* in prosthetic device infection. Previous work demonstrated that *S*. *aureus* strains isolated from invasive disease often possess both *fnbA* and *fnbB* [14]. We show here that the absence of *fnbB* does not prevent *S*. *aureus* from causing PJI, as 20% of the 27 isolates associated with PJI lacked *fnbB* by PCR. Similarly, the presence of *fnbB* was not associated with orthopedic device infection. This result is consistent with a past study that similarly found no association between the presence of *fnbB* and orthopedic implant infections [32], though the comparator group in this prior study was *S*. *aureus* isolates that caused infections in persons without an orthopedic implant.

The PJI and PJU groups also had a similar mean fibronectin binding capacity. While the fibronectin binding assay is arguably a crude tool of measurement, it is a well-established platform and has previously detected differences in fibronectin binding capacity [15,16]. The ability to form biofilm was also similar in the PJI and PJU groups.

Taken together, these results suggest that SNPs within *fnbA* and *fnbB* do not influence fibronectin binding, biofilm formation, or clinical outcome (PJI vs. PJU). These results stand in stark contrast to those we found in CDI as well as a recent study of *S*. *aureus* that cause infective endocarditis [33]. This suggests that additional factors in the environment surrounding cardiac devices, but not arthroplasties, contribute to the link between *fnbA* and *S*. *aureus* CDI.

This study has limitations. First, only *fnbA* and *fnbB* were examined. There are a variety of other MSCRAMMs beyond the scope of this study that would be of interest to sequence, such as sialoprotein-binding protein (*bbp*), clumping factor A (*clfA)*, clumping factor B (*clfB)*, collagen binding adhesion (*cna)*, or elastin-binding protein (*ebpS*). Moreover, the biological characteristic of redundancy is well known in *S*. *aureus*, making more than one mechanism possible. Second, none of the isolates lacked *fnbA*, making it impossible to assess the comparative importance of *fnbA* presence in the pathogenesis of *S*. *aureus* arthroplasty infection. Third, the growth media conditions in the assays (biofilm-forming capacity, fibronectin-binding capacity, quantitative PCR, Western ligand binding assay) differed slightly, which complicates direct comparison of assay results.

However, we conclude that genetic variation in *fnbA* and *fnbB* is not uniformly linked to the pathogenesis of foreign device infections. Instead, there appears to be a link between the physiological environment of the indwelling device, such as location, presence of a fibrin sheath, or device material, and the initiation of *S*. *aureus* prosthesis infection. Future studies are being performed to unravel this significant complication of medical progress.

## Supporting Information

S1 Fig Scatter plot of fibronectin binding capacity versus biofilm formation. Fibronectin binding capacity was normalized to that of control strain *S*. *aureus* 8325–4, and biofilm formation was normalized to that of *S*. *aureus* UAMS-1.
(TIF)

## References

[1] Peersman G, Laskin R, Davis J, Peterson M (2001) Infection in total knee replacement: a retrospective review of 6489 total knee replacements. Clin Orthop Relat Res 392: 15–23. 11716377

[2] Barberan J, Aguilar L, Carroquino G, Gimenez MJ, Sanchez B, Martinez D, et al (2006) Conservative treatment of staphylococcal prosthetic joint infections in elderly patients. Am J Med 119: 993e997–910. 10.1016/j.amjmed.2006.03.036 17071171

[3] Padera RF (2006) Infection in ventricular assist devices: the role of biofilm. Cardiovasc Pathol 15: 264–270. 16979033 10.1016/j.carpath.2006.04.008

[4] Vergara-Irigaray M, Valle J, Merino N, Latasa C, Garcia B, Ruiz de Los Mozos I, et al (2009) Relevant role of fibronectin-binding proteins in *Staphylococcus aureus* biofilm-associated foreign-body infections. Infect Immun 77: 3978–3991. 10.1128/IAI.00616-09 19581398 PMC2738049

[5] Geoghegan JA, Monk IR, O'Gara JP, Foster TJ (2013) Subdomains N2N3 of fibronectin binding protein A mediate *Staphylococcus aureus* biofilm formation and adherence to fibrinogen using distinct mechanisms. J Bacteriol 195: 2675–2683. 10.1128/JB.02128-12 23564165 PMC3676058

[6] O'Neill E, Humphreys H, O'Gara JP (2009) Carriage of both the *fnbA* and *fnbB* genes and growth at 37 degrees C promote FnBP-mediated biofilm development in meticillin-resistant *Staphylococcus aureus* clinical isolates. J Med Microbiol 58: 399–402. 10.1099/jmm.0.005504-0 19273632

[7] McCourt J, O'Halloran DP, McCarthy H, O'Gara JP, Geoghegan JA (2014) Fibronectin-binding proteins are required for biofilm formation by community-associated methicillin-resistant *Staphylococcus aureus* strain LAC. FEMS Microbiol Lett 353: 157–164. 10.1111/1574-6968.12424 24628034

[8] Casillas-Ituarte NN, Lower BH, Lamlertthon S, Fowler VG Jr., Lower SK (2012) Dissociation rate constants of human fibronectin binding to fibronectin-binding proteins on living *Staphylococcus aureus* isolated from clinical patients. J Biol Chem 287: 6693–6701. 10.1074/jbc.M111.285692 22219202 PMC3307253

[9] Lower SK, Lamlertthon S, Casillas-Ituarte NN, Lins RD, Yongsunthon R, Taylor ES, et al (2011) Polymorphisms in fibronectin binding protein A of *Staphylococcus aureus* are associated with infection of cardiovascular devices. Proc Natl Acad Sci U S A 108: 18372–18377. 10.1073/pnas.1109071108 22025727 PMC3215016

[10] Hos NJ, Rieg S, Kern WV, Jonas D, Fowler VG, Higgins PG, et al (2015) Amino acid alterations in fibronectin binding protein A (FnBPA) and bacterial genotype are associated with cardiac device related infection in *Staphylococcus aureus* bacteraemia. J Infect 70: 153–159. 10.1016/j.jinf.2014.09.005 25246358

[11] Kaasch AJ, Fowler VG Jr., Rieg S, Peyerl-Hoffmann G, Birkholz H, Hellmich M, et al (2011) Use of a simple criteria set for guiding echocardiography in nosocomial *Staphylococcus aureus* bacteremia. Clin Infect Dis 53: 1–9. 10.1093/cid/cir320 21653295 PMC3149212

[12] Kaasch AJ, Barlow G, Edgeworth JD, Fowler VG Jr., Hellmich M, Hopkins S, et al (2014) *Staphylococcus aureus* bloodstream infection: A pooled analysis of five prospective, observational studies. J Infect 68: 242–251. 10.1016/j.jinf.2013.10.015 24247070 PMC4136490

[13] Nienaber JJ, Sharma Kuinkel BK, Clarke-Pearson M, Lamlertthon S, Park L, Rude TH, et al (2011) Methicillin-susceptible *Staphylococcus aureus* endocarditis isolates are associated with clonal complex 30 genotype and a distinct repertoire of enterotoxins and adhesins. J Infect Dis 204: 704–713. 10.1093/infdis/jir389 21844296 PMC3156104

[14] Peacock SJ, Day NP, Thomas MG, Berendt AR, Foster TJ (2000) Clinical isolates of *Staphylococcus aureus* exhibit diversity in fnb genes and adhesion to human fibronectin. J Infect 41: 23–31. 10942636 10.1053/jinf.2000.0657

[15] Sharma-Kuinkel BK, Mann EE, Ahn JS, Kuechenmeister LJ, Dunman PM, Bayles KW (2009) The *Staphylococcus aureus* LytSR two-component regulatory system affects biofilm formation. J Bacteriol 191: 4767–4775. 10.1128/JB.00348-09 19502411 PMC2715716

[16] Beenken KE, Blevins JS, Smeltzer MS (2003) Mutation of *sarA* in *Staphylococcus aureus* limits biofilm formation. Infect Immun 71: 4206–4211. 12819120 10.1128/IAI.71.7.4206-4211.2003 PMC161964

[17] Thurlow LR, Hanke ML, Fritz T, Angle A, Aldrich A, Williams SH, et al (2011) *Staphylococcus aureus* biofilms prevent macrophage phagocytosis and attenuate inflammation *in vivo* . J Immunol 186: 6585–6596. 10.4049/jimmunol.1002794 21525381 PMC3110737

[18] Boles BR, Horswill AR (2008) Agr-mediated dispersal of *Staphylococcus aureus* biofilms. PLoS Pathog 4: e1000052 10.1371/journal.ppat.1000052 18437240 PMC2329812

[19] Buck AW, Fowler VG Jr., Yongsunthon R, Liu J, DiBartola AC, Que YA, et al (2010) Bonds between fibronectin and fibronectin-binding proteins on *Staphylococcus aureus* and *Lactococcus lactis* . Langmuir 26: 10764–10770. 10.1021/la100549u 20218549 PMC2893610

[20] Benjamini Y, Hochberg Y (1995) Controlling the False Discovery Rate—a Practical and Powerful Approach to Multiple Testing. Journal of the Royal Statistical Society Series B-Methodological 57: 289–300.

[21] Herrmann M, Vaudaux PE, Pittet D, Auckenthaler R, Lew PD, Schumacher-Perdreau F, et al (1988) Fibronectin, fibrinogen, and laminin act as mediators of adherence of clinical staphylococcal isolates to foreign material. J Infect Dis 158: 693–701. 3171224 10.1093/infdis/158.4.693

[22] Hoshal VL Jr., Ause RG, Hoskins PA (1971) Fibrin sleeve formation on indwelling subclavian central venous catheters. Arch Surg 102: 353–358. 5553297 10.1001/archsurg.1971.01350040115023

[23] Mehall JR, Saltzman DA, Jackson RJ, Smith SD (2002) Fibrin sheath enhances central venous catheter infection. Crit Care Med 30: 908–912. 11940768 10.1097/00003246-200204000-00033

[24] Vaudaux P, Pittet D, Haeberli A, Huggler E, Nydegger UE, Lew DP, et al (1989) Host factors selectively increase staphylococcal adherence on inserted catheters: a role for fibronectin and fibrinogen or fibrin. J Infect Dis 160: 865–875. 2809259 10.1093/infdis/160.5.865

[25] Puleo DA, Nanci A (1999) Understanding and controlling the bone-implant interface. Biomaterials 20: 2311–2321. 10614937 10.1016/s0142-9612(99)00160-x

[26] Tung H, Guss B, Hellman U, Persson L, Rubin K, Ryden C (2000) A bone sialoprotein-binding protein from *Staphylococcus aureus*: a member of the staphylococcal Sdr family. Biochem J 345 Pt 3: 611–619. 10642520 PMC1220796

[27] Campoccia D, Speziale P, Ravaioli S, Cangini I, Rindi S, Pirini V, et al (2009) The presence of both bone sialoprotein-binding protein gene and collagen adhesin gene as a typical virulence trait of the major epidemic cluster in isolates from orthopedic implant infections. Biomaterials 30: 6621–6628. 10.1016/j.biomaterials.2009.08.032 19758694

[28] Vancraeynest D, Hermans K, Haesebrouck F (2004) Genotypic and phenotypic screening of high and low virulence *Staphylococcus aureus* isolates from rabbits for biofilm formation and MSCRAMMs. Vet Microbiol 103: 241–247. 15504595 10.1016/j.vetmic.2004.09.002

[29] Wiggins MJ, Wilkoff B, Anderson JM, Hiltner A (2001) Biodegradation of polyether polyurethane inner insulation in bipolar pacemaker leads. J Biomed Mater Res 58: 302–307. 11319745 10.1002/1097-4636(2001)58:3<302::aid-jbm1021>3.0.co;2-y

[30] Haqqani HM, Mond HG (2009) The implantable cardioverter-defibrillator lead: principles, progress, and promises. Pacing Clin Electrophysiol 32: 1336–1353. 10.1111/j.1540-8159.2009.02492.x 19796348

[31] Katti KS (2004) Biomaterials in total joint replacement. Colloids Surf B Biointerfaces 39: 133–142. 15556342 10.1016/j.colsurfb.2003.12.002

[32] Arciola CR, Campoccia D, Gamberini S, Baldassarri L, Montanaro L (2005) Prevalence of cna, fnbA and fnbB adhesin genes among Staphylococcus aureus isolates from orthopedic infections associated to different types of implant. FEMS Microbiol Lett 246: 81–86. 15869965 10.1016/j.femsle.2005.03.035

[33] Xiong YQ, Sharma-Kuinkel BK, Casillas-Ituarte NN, Fowler VG Jr., Rude T, DiBartola AC, et al (2015) Endovascular infections caused by methicillin-resistant Staphylococcus aureus are linked to clonal complex-specific alterations in binding and invasion domains of fibronectin-binding protein A as well as the occurrence of fnbB. Infect Immun 83: 4772–4780. 10.1128/IAI.01074-15 26416903 PMC4645417

