# Supplementary figures and images for: Polymorphisms in Fibronectin Binding Proteins A and B among Staphylococcus aureus Bloodstream Isolates Are Not Associated with Arthroplasty Infection
Source: PLoS One. 2015 Nov 25;10(11):e0141436. doi: 10.1371/journal.pone.0141436 (PMC4659655; doi:10.1371/journal.pone.0141436)

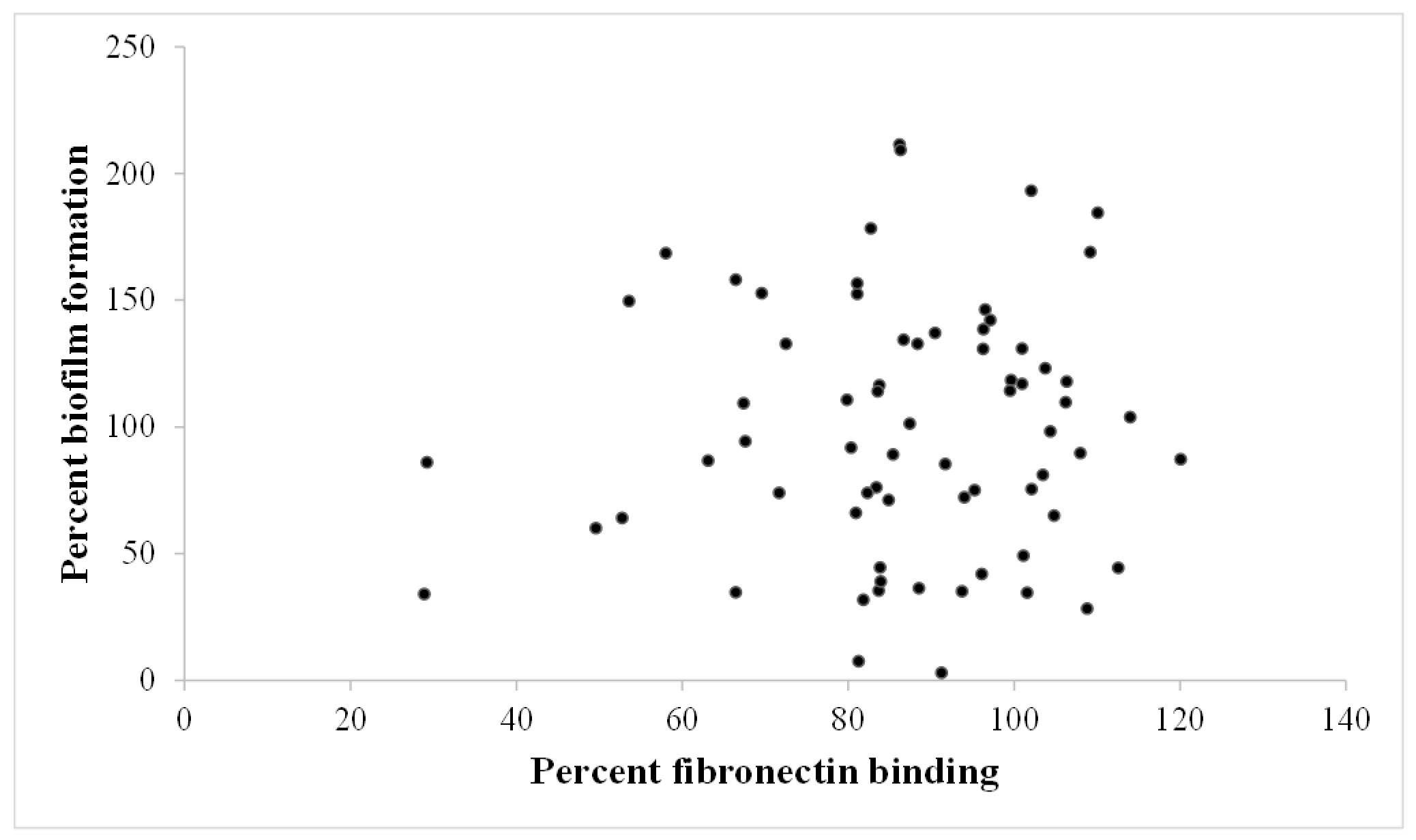

Supplement: S1 Fig — Fibronectin binding capacity was normalized to that of control strain S. aureus 8325–4, and biofilm formation was normalized to that of S. aureus UAMS-1. (TIF) [file pone.0141436.s001.tif]
